# The patients awareness and medication adherence among high-risk stroke patients admitted in a tertiary level hospital in Qatar: A cross-sectional study

**DOI:** 10.5339/qmj.2025.49

**Published:** 2025-07-05

**Authors:** Nesiya Hassan, Bejoy Varghese, Annamma Jose, Emad Salem Ajlouni, Nisha George, Pacifico Jr. Gamarcha Gellego, Rida Moh’d Odeh A.M. Al-Balawi, Rajvir Singh

**Affiliations:** 1 Nursing and Midwifery Research Department, Hamad Medical Corporation, Doha, State of Qatar; 2 Department of Nursing Education, In-patient Services, Hamad General Hospital, Doha, State of Qatar; 3 Medical Department, In-patient services, Hamad General Hospital, Doha, State of Qatar; 4 Neurosciences and Surgical Sub-Specialty Department, In-patient services, Hamad General Hospital, Doha, State of Qatar; 5 Academic Health System, Hamad Medical Corporation, Doha, State of Qatar *Email: bejoy1987@gmail.com

**Keywords:** Medication adherence, stroke, prevention, medication compliance, knowledge, Qatar

## Abstract

**Introduction:**

The burden of stroke is increasing in Qatar, similar to many other countries in the world. The prevalence of stroke risk factors plays an important role in the burden of stroke in Qatar.

**Objective:**

This study aims to assess the stroke knowledge, recognition, prevention, and medication adherence among high-risk patients.

**Methods:**

This was a cross-sectional study involving high-risk patients admitted to the inpatient medical unit through a paper-based survey using four adopted scales Stroke knowledge test, Stroke Recognition Questionnaire, stroke prevention awareness, and Medication Adherence Rating Scale tool. Two hundred ninety-nine completed responses from the participants were used for analysis.

**Results:**

The 41–50-year-old age group was most represented (33.11%) with a median age of 49 years. The male-female ratio was approximately 3:1 and 30.10% of the participants held a graduate degree or higher.The majority of the patients (41.47%) are obese (BMI > 30) and overweight (31.10%). The mean stroke knowledge was 38.01 ± 15.78, while the recognition and prevention domains had mean scores of 67.85 ± 10.85 and 77.55 ± 27.63 respectively. The medication adherence group has a statistically significant association with stroke recognition and prevention domains with a mean score of 72.14 ± 12.34 (*p* = 0.000) and 88.87 ± 24.75 (*p* = 0.000) respectively.

**Conclusion:**

Participants’ awareness regarding stroke knowledge was low compared to recognition and preventive measures. Stroke awareness positively correlates with medication adherence among high-risk patients admitted to the inpatient unit.

## INTRODUCTION

Stroke is the leading cause of morbidity and the second top reason for mortality worldwide.^1^ The Global Stroke Fact Sheet in 2022 revealed that the lifetime risk of developing a stroke has increased by 50% over the last 17 years. At present, 1 in 4 people over the age of 25 are estimated to have a stroke in their lifetime. The incidence of stroke is reported globally at around 1.2 million each year, and over 101 million people are currently living with disabilities due to stroke. Moreover, 86% of deaths due to stroke occur in lower and lower-middle-income countries.^2^ The multinational Biorepository of DNA in Stroke (BRAINS) study revealed that the annual incidence rate of stroke in Qatar was 58 per 100,000 per year, with a mortality rate of 9.17 per 100,000 per year. Previous research has identified hypertension, diabetes, and dyslipidemia as the most common risk factors for stroke, with a higher prevalence among Qatari natives compared to expatriates. Understanding these disparities is essential for assessing stroke awareness and medication adherence behaviors among high-risk patients.

## REVIEW OF LITERATURE

According to the American Stroke Association, modifiable risk factors for stroke include hypertension, diabetes mellitus, obesity, hypercholesterolemia, coronary artery disease, and atrial fibrillation.^5^ Since our study focuses on high-risk inpatients, evaluating their awareness of these risk factors alongside their medication adherence is critical in developing targeted interventions. Modifying or controlling stroke risk factors through motivating and influencing patient behaviors is the most effective way to reduce stroke morbidity and mortality. Public knowledge about stroke and its prevention is an important component of the success of the stroke prevention program.^6^ A recent study in the Middle East found that participants had a high level of knowledge and a positive attitude towards stroke.^7^ Many international studies have reported similar results.^8–11^ However, there is inconsistency regarding these findings. Many studies reported a low level of knowledge regarding identifying the signs and symptoms, risk factors, and appropriate responses during stroke emergencies.^12–15^ This underscores the significance of community-based education regarding various aspects of stroke and emphasizes the need for stroke prevention programs. Several factors, including gender, age, educational background, and personal or family history of stroke influence awareness of stroke prevention. Stroke prevention knowledge is higher among male participants compared to females. Similarly, older people have a better understanding of stroke signs and symptoms.^16^ Moreover, people who had a history of stroke had a higher score on preventive behaviors than those without risk.^17^ Furthermore, formal education was a strong predictor of participants’ awareness of stroke signs and symptoms, risk factors, and prevention strategies.^18^

The relationship between medication adherence and stroke outcomes highlights a pivotal area of research. Adherence to a medication regimen is the best preventive strategy against stroke. Medication adherence emerges as a basis for preventing the initial occurrence of stroke and mitigating the risk of recurrence.^19^ However, patients’ knowledge and attitudes act as strong predictors of their medication adherence. Kvarnström et al.; reported that major barriers to medication adherence include patient-specific factors, characteristics of drug therapies, issues of the healthcare system, and the role and functions of healthcare professionals.^20^ Furthermore, disability, decreased cognitive function, polypharmacy, concerns about treatment, and a history of stroke are commonly identified patient-related factors of non-adherence to medications.^21^ The risk of stroke may escalate with non-adherence to medication and can deteriorate patient outcomes significantly. A recent study in Indonesia proved that stroke knowledge was independently associated with the participant’s medication adherence rate.^22^

In 2014, our Organization launched its stroke services, prominently including the FAST Program as a major highlight. Recent statistics show that more than 16,000 patients suspected of having acute stroke have received treatment in Stroke Service. This data indicates that quite a large number of patients are getting stroke treatment within the region. There is a paucity of studies to assess the stroke awareness of the public in Qatar. This gap in knowledge underscores the importance of assessing the level of stroke awareness, especially among patients who are at high risk of developing stroke. The present study aims to compare the stroke awareness and medication adherence behaviors of high-risk patients admitted to medical inpatient units. Also, this study explores the association between demographic variables and stroke knowledge, recognition, prevention, and medication adherence.

## METHODS

### Design

This was a cross-sectional study to evaluate stroke awareness and medication adherence among high-risk patients admitted to medical inpatient units. This study collected information on the participants’ stroke knowledge, ability to recognize their signs and symptoms, risk factors, preventive measures, and medication adherence through a self-administered survey.

### Research questions

The following research questions were focused in this study:

- What is the stroke awareness among high-risk stroke patients admitted to inpatient units?
- Is there a relationship between stroke awareness and medication adherence among high-risk stroke patients?
- Are there any significant differences among the demographical characteristics of the participants with regard to their stroke awareness and medication adherence?

### Sample size

The sample size was calculated based on the prevalence of knowledge regarding stroke at 76.6%.^6^ The study required 280 subjects at a 95% confidence interval, and 5% precision, and with the consideration of a 5% incomplete questionnaire, the final sample size was 294. The completed responses from 299 participants were taken for final analysis.

### Setting and sample

The study was conducted at one of the largest tertiary-level healthcare facilities in Qatar. The facility consists of a monthly admission of 1,200 patients in medical inpatient units. The population was composed of high-risk patients admitted to medical inpatient units during the index period.

### Inclusion/exclusion criteria

The study included adult male and female patients admitted to the medical inpatient unit who had a history of smoking or medical conditions such as hypertension, diabetes mellitus, high LDL, arterial malformations, brain aneurysms, or cardiovascular diseases, including coronary artery disease, atrial fibrillation, heart valve disease, or carotid artery disease.

The study excluded patients with a history of stroke, significant physical, communication impairment or mental health problems that interfered their ability to read or comprehend the questionnaire. Patients who were unable to read or understand the questionnaire in English, Arabic, or Hindi were also excluded from the study.

**Patient recruitment and consent process**

Eligible patients admitted to the medical inpatient unit were identified by the research team based on the inclusion and exclusion criteria. The research team approached the patients, explained the study’s purpose, procedures, and potential benefits, and provided an information sheet with detailed study information. Patients were given up to 24 hours to decide on participation. To maintain confidentiality, no personal identifiers were collected, and participation was entirely voluntary, with no follow-up required after data collection.

### Data collection

The data was gathered in 3 months, between October and December 2023. The study collected information regarding participants’ stroke awareness and medication adherence through a self-administered questionnaire. Patients who met the inclusion criteria and had been admitted to the medical unit during the index period form the sample population. Eligible individuals were invited to participate in the research by the research team. This study doesn’t collect any personal identifier or information from the patient’s electronic medical record, so informed consent was not obtained. The principal investigator regularly collected the completed questionnaire from the research lockers of each unit.

Four validated questionnaires were used in this study to comprehensively assess stroke knowledge, recognition, prevention, and medication adherence among high-risk patients. While we acknowledge that completing multiple questionnaires may be burdensome for some participants, the total estimated completion time was approximately 20–30 minutes, which was deemed reasonable for an inpatient setting. Participants were also given the option to take breaks while completing the survey to minimize fatigue. Using these tools allowed for a more in-depth understanding of the study variables, supporting the study’s objectives.

**Categorization of stroke awareness levels**

The participants were categorized into three based on their cutoff score. The participants with less than 50% scored as low, 51-70% as moderate and above 70% as high in three aspects of stroke awareness namely stroke knowledge, stroke recognition and stroke prevention.

### Instruments

The study collected the information by using four tools: Stroke knowledge test (SKT), Stroke Recognition Questionnaire (SRQ), Stroke prevention questionnaire, and Medication Adherence Rating Scale (MARS). The demographic part collected information from the participants, including age, gender, educational status, BMI, nationality, smoking, family history of stroke, medical insurance, and occupation. The SKT was developed and validated by Dr. Karen Sullivan^23^ and consists of 20 multiple-choice questions, with each correctly identified response carrying one point. The total possible knowledge score is 20, with a minimum score of zero. The SKT has demonstrated adequate internal consistency reliability (**α** = 0.65) and strong test-retest reliability (r = 0.82).^23^ The SRQ, developed by Ennen and Zerwic, is a reliable instrument for assessing knowledge of stroke risk factors and warning signs.^24^ The SRQ consists of 20 items, with 10 questions covering stroke risk factors and 10 addressing non-risk factors. The Content Validity Index of the SRQ was reported as 0.95, with subscale reliability scores of 0.70 for stroke risk factors and 0.81 for stroke symptoms.^24^ The maximum score of SRQ is 20, and the minimum score is zero. The stroke prevention awareness questions were adopted from the American Stroke Association’s^25^ public stroke prevention program. This involves 12 statements concerning preventive behaviors, which were used to assess awareness through yes or no responses (where 1 represents yes and 0 represents no). Each correct statement carries one point, with a maximum possible score of 12 and a minimum score of zero. The MARS,^26^ which was developed by Dr. K. Thompson, consists of 10 statements regarding the participant’s behavior. This multidimensional instrument describes three dimensions: medication adherence behavior (Q1–Q4), attitude towards taking medication (Q5–Q8), and negative side effects and attitudes (items Q9–Q10). The total score ranges from 0 to 10, with a higher score indicating better adherence, and items 7 and 8 are reversely coded. The internal consistency of MARS Cronbach’s alpha was 0.75, and the test-retest reliability was MARS 0.76. Respondents scoring 8 and above were categorized as the “medication adherence group,” while those scoring below this threshold were classified as the non-adherence group.

### Statistical analysis

Descriptive statistics were used to summarize and determine the sample characteristics and distribution of participants’ data. The normally distributed data and results were reported with a mean and standard deviation (SD). The knowledge of stroke, stroke recognition, prevention, and medication adherence were calculated, to sum up, all the questions for different domains. Categorical data was summarized using frequencies and proportions. Associations between two or more quantitative data variables were assessed using the Chi-square (**χ2**) test. Quantitative data between the two or more independent groups was analyzed using the unpaired t-test, or ANOVA. All *p* values presented were two-tailed, and *p* values <0.05 were considered statistically significant. In this study, stroke awareness was calculated by the sum of scores in stroke knowledge, recognition, and prevention. The knowledge, recognition of warning signs, and prevention of stroke scores were transformed to a scale ranging from 0 to 100. The total mean score was calculated by multiplying the domain index score by 100. All statistical analyses were done using the statistical package SPSS 29.0.

## RESULTS

### Sample characteristics

The complete responses from the two hundred ninety-nine patients were taken for analysis. The 41–50-year-old age group was most represented (33.11%), followed by the 51–60-year-old age group (25.42%). The median age was 49 years, which ranges from 19 to 80 years. The male-female ratio was approximately 3:1, and 30.10% of the participants had graduated and above-level education, followed by higher secondary (27.76%) and secondary (27.42%) categories. The majority of the participants were from Asia and the Middle East, accounting for 59.53% and 30.10%, respectively. Interestingly, the majority of the participants are nonsmokers (47.83%), but 25.75% agreed with their habit of daily smoking. Furthermore, the majority of the patients (41.47%) are obese (BMI > 30) and overweight (31.10%), and 29 patients don’t have any preexisting medical conditions. Most of the participants have hypertension and diabetes, constituting 66.22% and 52.51%, respectively. Similarly, only 72 patients had a family history of stroke, and most of the patients (60.54%) were not covered by any medical insurance. Table 1 displays the sociodemographic characteristics of the participants.

### Total stroke awareness score among participants

The stroke knowledge domain, the mean score was 38.01 (SD 15.78), while the recognition and prevention domains had mean scores of 67.85 (SD 10.85) and 77.55 (SD 27.63). Most of the participants (56.22%) held moderate scores in stroke recognition, in contrast to 81.27% with low stroke knowledge. Surprisingly, 64.5% of the patients have a high level of knowledge about stroke prevention measures (Table 2).

### Factors affecting stroke knowledge, recognition, prevention, and medication adherence among high-risk patients

Table 3 illustrates the factors associated with stroke knowledge, recognition, prevention, and medication adherence. The knowledge score was significantly higher in participants with graduate and above education (44.67 ± 14.66, *p* = 0.000), as compared with primary level (29.09 ± 15.45), secondary level (36.71 ± 15.56), and higher secondary (36.81 ± 14.60) level of education. A similar pattern was observed in the medication adherence domain, patients with graduate and above education have a higher mean score (62.89 ± 21.32, *p* = 0.045) compared with other education categories of patients. These findings suggest that education has a key role in enhancing the knowledge of participants, which in turn promotes their adherence to medication. The participants with a family history of stroke hold higher knowledge scores (42.43 ± 14.89, *p* = 0.006), prevention scores (86.69 ± 24.64), and medication adherence scores (69.58 ± 19.60, *p* = 0.000) compared with others. These results suggest that family history of stroke is a key predictor in improving participants knowledge of stroke and preventive measures, and, moreover, enhancing their medication adherence. Interestingly, the mean knowledge score is higher among participants in the obese category (42.14 ± 14.22, *p* = 0.001) compared with the overweight (35.97 ± 16.07) and ideal body weight (35.07 ± 16.70) groups. Whereas medication adherence score was higher among the overweight group (62.80 ± 20.45, *p* = 0.008) compared with other categories of patients. This indicates that obese and overweight patients were more concerned with improving their knowledge and medication adherence. Gender is statistically significant in stroke recognition, prevention, and medication adherence. The female patients held a higher mean score in recognition (70.04 ± 11.39, *p* = 0.024), prevention (86.44 ± 25.55, *p* = 0.002), and medication adherence (63.86 ± 24.57, *p* = 0.027). These findings provide evidence that females are more concerned about health-related matters and its potential consequences. Age is statistically significant in stroke prevention and medication adherence subdomains. The higher mean score of stroke prevention was found among participants above 60 years (88.00 ± 22.04, *p* = 0.014). Similarly, the medication adherence score was higher among the above-60-year-old group (65.93 ± 21.82, *p* = 0.025) compared with other age groups. These findings support the idea that age is a strong influencing factor in maintaining participant medication adherence and their ability to follow stroke prevention measures. A similar pattern followed among patients in the smoking group and non-smoking categories. Non-smokers had a higher score (63.50 ± 21.79, *p* = 0.004) compared with other categories, which indicated that non-smokers are more concerned about their health and the consequences of nonadherence to medication regimens.

Table 4 illustrates the stroke knowledge, recognition, and prevention scores among medication adherence and non-adherence groups. The medication adherence group has a statistically significant association with stroke recognition and prevention domains, with a mean score of 72.14 ± 12.34 (*p* = 0.000) and 88.87 ± 24.75 (*p* = 0.000), respectively. Table 5 shows the correlation between stroke awareness and medication adherence among the participants. The Pearson correlation shows the relationship between stroke awareness and medication adherence is significant (r = 0.224, *p* = 0.000), which indicates both are correlated with a weak positive association with the variables.

## DISCUSSION

This study is one of the first studies in Qatar to investigate stroke knowledge, recognition of warning signs, prevention, and medication adherence among high-risk patients in Qatar. We assessed stroke awareness with associated factors such as education, age, gender, education, past medical history, BMI, family history of stroke, and insurance status of the respondents. Generally, the overall stroke awareness of the high-risk patients was at a moderate level. These findings provide concrete evidence to support implementing interventions to enhance awareness among patients who are at high risk of developing stroke. This can help reduce the incidence of stroke in Qatar and enhance the quality of life for high-risk patients.

The participants of the current study had extremely low knowledge regarding stroke, which is consistent with the previous studies conducted in Asia.^9^ However, there is inconsistency regarding this knowledge. The studies conducted in the Middle East and Africa supported that the participants had good knowledge regarding stroke. Approximately half of the participants correctly identified that stroke occurs when the blood supply to the brain is blocked. These findings in the current study are higher when compared with other studies conducted in Ireland and Nigeria. Less than one-third of the patients correctly identified the transient ischemic attack (TIA) and its symptoms, which indicates that the participants were unable to identify the TIA as the common precursor to a stroke. In terms of immediate response to stroke, more than two-thirds of participants agreed that they would call an ambulance immediately. However, these findings are in contrast with other studies that stated a low level of awareness in seeking urgent medical care.Many factors can influence the knowledge level of the participants. Our study revealed that the educational level, family history of stroke, and BMI of the individuals potentially had a higher level of knowledge compared to other participants. Whereas in previous studies was evident that age, gender, marital status, and educational level were correlated with stroke knowledge.

In the present study, the participant’s knowledge of warning signs for stroke was moderate. According to the cut-off used in this study, half of the participants had a moderate level of knowledge, while the rest were familiar with the majority of the warning signs of stroke. This was in contrast with the findings that have been reported from other study.^13^ The most commonly identified sign of stroke was slurred speech, numbness on one side of the face, weakness on one side of the body, and confusion, which is similar to previous study.^7^ However, most of the participants reported incorrectly the warning signs such as double vision and sudden onset of severe headache, consistent with the previous study.^29^ Hence, high-risk individuals need to be educated more extensively about the warning signs of stroke, which have significant implications for obtaining treatment promptly.

The knowledge regarding risk factors for stroke was found to be moderate, similar to the findings of other international studies.^7^ Most of the participants recognized that high blood pressure, alcohol intake, high cholesterol, and smoking were the major risk factors for stroke. However, only one-half of the respondents knew the risk of developing stroke among individuals with diabetes. These findings are consistent with the previous studies. Additionally, one-third of the participants are aware that obesity, a lack of physical activities, and a previous history of myocardial infarction can lead to the incidence of stroke, which aligns with a previous study. Surprisingly, very few participants are aware that neck vein diseases, irregular heartbeats, and varicose veins can progress to stroke.

Many factors can influence the knowledge of recognizing warning signs. The current study shows that gender has an association with stroke recognition. The female participants had better stroke recognition scores compared with males, whereas, many international studies identified that age, marital status,^31^ occupation,^9^ family history of stroke, education, and gender^5^ regions^32^ had an association with the participant’s knowledge about the recognition of warning signs.

Prevention of stroke may be more important than awareness of stroke symptoms. The growing global burden of disability attributed to stroke, rising costs of treatments, compromising the patient’s quality of life, and productive outcome. Pandian et al.; recommended three levels of prevention, including primordial prevention, primary prevention (preventing the onset of disease), and preventing the recurrence of stroke through secondary prevention.^33^ Primordial prevention aims to prevent the development of risk factors, and primary prevention is mainly focused on individual-level strategies for those who are at risk of stroke. In the current study, stroke prevention knowledge obtained the highest score compared with stroke knowledge and recognition, which is consistent with previous studies from the Middle East.^12^ Avoiding smoking and secondary smoking, reducing consumption of high-salt and fatty foods, and engaging in physical activity as preventive strategies had 80% agreement with the participants. Similarly, control of overweight, reducing alcohol intake, appropriate treatment of hypertension, and medication adherence had higher scores among three-quarters of the respondents, similar findings from previous studies.^7^ The present study findings show that most of the respondents demonstrated a high level of awareness regarding stroke prevention. Stroke prevention was correlated with the study variables, including age, gender, and family history of stroke.

In our study, respondents above 60 years old showed a statistically significantly higher score for stroke prevention than those below this age group. Females and individuals with a family history of stroke had higher scores in stroke prevention compared to male participants and those without such a family history, respectively. Whereas a previous study reported that education, occupation,^12^ and gender^34^ were significantly associated with higher stroke prevention scores. Knowledge is key for changing behaviors; however, addressing the underlying factors in changing behaviors is vital to know their practice towards stroke prevention.

Medication adherence is an important predictor of preventing secondary stroke.^33^ Many factors can influence adherence behaviors, including side effects of medication, negative public opinion about medicine, swallowing difficulties, the burden of disease, and financial status.^35^ However, factors promoting medication adherence may include education on the importance of medication compliance, positive experiences of taking medications, and the availability of modifications to manage side effects.^35^

The current study shows that most of the participants had nonadherence to their daily medications. This finding was consistent with previous studies^17^ that reported a high prevalence of medication nonadherence. However, In the present study, respondents above 60 years old reported higher medication adherence compared to younger participants. Additionally, females exhibited higher medication adherence scores compared to males. Other factors positively associated with medication adherence included a family history of stroke, non-smoking status, and BMI levels. Conversely, a study by Shankari et al. found that young age and higher economic status were associated with non-adherence to medication.

Furthermore, patients in this study were treated with direct oral anticoagulants which are recognized as the first-line therapy for atrial fibrillation, particularly in individuals with additional stroke risk factors. Numerous studies emphasize the critical role of anticoagulants in stroke prevention. For instance, a systematic review of randomized controlled trials involving atrial fibrillation patients demonstrated that appropriately adjusted doses of oral anticoagulants are extraordinarily effective in preventing strokes.^37^ Additionally, a retrospective cohort analysis using data from the US Market Scan claims databases provided compelling evidence that higher adherence rates—measured by lower medication discontinuation—significantly lowered the incidence of strokes.^38^ Therefore, ensuring strict adherence to anticoagulant therapy is not just important; it is essential for maximizing treatment benefits and achieving optimal efficacy.

The current study evidently shows a positive correlation between stroke awareness and medication adherence.

The findings of nonadherence to medication among high-risk patient populations have significant implications for clinical practice. The healthcare team needs to pay more attention to implementing appropriate age-specific strategies to improve patients’ awareness regarding stroke and the importance of medication adherence to prevent the incidence of stroke. The intervention should focus on modifying behaviors concerning physical activity, lifestyle, and dietary modifications. Hence, it can help patients lead a high-quality life and prevent stroke.

### Strengths and limitations

To our knowledge, this is the first study to assess stroke awareness among high-risk patient groups in Qatar. The sample consisted of admitted patients in the medical inpatient unit with a high-risk medical history or smoking habit, increasing the likelihood that the results represent the awareness of the high-risk patient community in Qatar. Additionally, conducting a paper-based survey allowed patients sufficient time to complete the questionnaire during their hospital admission, reducing potential response fatigue. The study utilized validated questionnaires to ensure the reliability of data collection.

However, this study has some limitations. While we included inpatients from one of the largest healthcare facilities in Qatar, other general hospitals under the same healthcare organization were not involved, limiting the generalizability of the findings. The study relied on self-reported data for medication adherence, which may have been influenced by recall bias and social desirability bias, despite efforts to assure participants of confidentiality. Additionally, patients with aphasia and cognitive impairments were excluded, which may have limited the representation of stroke survivors with communication challenges. The use of a non-probability sampling method introduces the possibility of selection bias, affecting the overall external validity of the results. Another potential limitation of this study is the presence of concomitant illnesses among stroke patients, which may act as confounding variables influencing stroke awareness, recognition, prevention behaviors, and medication adherence.

### Implications for practice

In light of our findings from the current study, we strongly recommend the Ministry of Public Health in Qatar to introduce targeted public education programs to enhance awareness regarding stroke across Qatar’s diverse populations. This program should emphasize on recognizing stroke signs and symptoms, preventive strategies, and awareness of risk factors. Additionally, hospitals and primary health centers can implement personalized counseling therapy focused on lifestyle modifications, such as dietary guidance and exercise regimens, which could be beneficial for the large group of high-risk patients affected by obesity and overweight. Establishing a routine follow-up program could be beneficial to promote medication adherence among high-risk groups. Furthermore, integrating a structured discharge planning guideline for high-risk patients including referral to the outpatient department, community services for stroke prevention, and medication adherence support could enhance long-term health outcomes.

## CONCLUSION

The findings from this study showed that high-risk patients who participated in this study had moderate knowledge of stroke awareness. In addition, most of the high-risk patients exhibited low medication adherence. Stroke knowledge independently correlates with medication adherence. The high-risk population may benefit from increased stroke awareness that promotes medication adherence. Long-term, this will help reduce the healthcare burden caused by stroke. According to our study findings, a multidisciplinary healthcare team including general practitioners, neurologists, nurses, dietitians, and pharmacists must focus on regularly educating all patients, particularly those at risk, about stroke, the recognition of warning signs, prevention, monitoring their risk factors, and the importance of medication compliance throughout their lifetime. Therefore, it would also be highly important to assess the knowledge level of the general population regarding awareness of stroke and its preventive measures.

## Acknowledgments

The author would like to extend appreciation to the nursing leadership of Hamad General Hospital for administrative support.

## Conflicts of interest

The authors have no conflicts of interest to declare.

## Funding

The authors declared that this study has been funded by the Medical Research Center (MRC).

## Ethical approval

The study received ethical approval from the Institutional Review Board (IRB) of Hamad Medical Corporation (HMC). It was carried out in adherence to the guidelines and principles set out in the Declaration of Helsinki and Good Clinical Practice (GCP) and in compliance with the laws and regulations governing research conducted by the Ministry of Public Health (MoPH) in Qatar. The study number is MRC-01–23-346.

## Authors’ contribution

NH: literature search, conception and design data analysis, and manuscript writing; BV: literature search, conception and design, data collection, and manuscript writing; RB, AJ, ES, PG, RM, and NG: data collection, drafting the manuscript, and peer review; RS: data analysis, manuscript writing, peer review and revising, and final approval. All the authors have read and approved the final manuscript.

## Figures and Tables

**Table 1.** Sociodemographic characteristics of the participant.

**Variables**	**Categories**	**Frequency % (N = 299)**
Age	<30	16 (5.35)
	31–40	49 (16.39)
	41–50	99 (33.11)
	51–60	76 (25.42)
	>60	59 (19.73)
Gender	Male	229 (76.59)
	Female	70 (23.41)
Qualification	Primary	44 (14.72)
	Secondary	82 (27.42)
	Higher Secondary	83 (27.76)
	Graduate and above	90 (30.10)
Nationality	Asian	178 (59.53)
	Africans	21 (7.02)
	Middle east	90 (30.10)
	Western	10 (3.34)
Smoking history	Occasionally	42 (14.05)
	Daily	77 (25.75)
	Never	143 (47.83)
	Stopped	37 (12.37)
BMI	<20	11 (3.68)
	20.1–25	71 (23.75)
	25.1–30	93 (31.10)
	>30	124 (41.47)
Past medical history	Hypertension	198 (66.22)
	Diabetic	157 (52.51)
	Atrial fibrillation	6 (2.01)
	High cholesterol	7 (2.34)
	Coronary heart disease	20 (6.69)
Family history of stroke	Yes	72 (24.08)
	No	227 (75.92)
Medical insurance	Yes	118 (39.46)
	No	181 (60.54)

**Table 2.** The number of items, total score, and the participant’s level of stroke knowledge, recognition, and prevention.

**Variable**	**Number of items**	**Total responses**	**Range of score**	**Total score%**	**Low**	**Moderate**	**High**
				**mean ± SD**	**(<50%)**	**(51–70%)**	**(>70%)**
Stroke knowledge	20	299	0–100	38.01 ± 15.78	81.27	17.73	1
Stroke recognition	40	299	0–100	67.85 ± 10.85	5.05	56.82	38.13
Stroke prevention	12	299	0–100	77.55 ± 27.63	20.07	15.38	64.55

**Table 3.** Factors associated with stroke knowledge, recognition, prevention, and medication adherence of the participants.

**Variables**	***N***	**Stroke knowledge (%)**	**Stroke recognition (%)**	**Stroke prevention (%)**	**Medication adherence (%)**
		**Mean (SD)**			
**Age**					
<30	16	35.93 (13.81)	63.91 (9.83)	80.69 (22.57)	51.88 (22.57)
31–40	49	35.20 (15.61)	66.89 (11.47)	74.71 (29.14)	58.98 (24.43)
41–50	99	39.29 (15.08)	67.45 (9.83)	76.45 (28.17)	55.56 (20.86)
51–60	76	39.54 (15.41)	68.42 (11.59)	72.04 (29.16)	59.61 (16.28)
>60	59	36.78 (17.95)	69.70 (11.22)	88.00 (22.04)	65.93 (21.82)
***p-value***		***0.472***	***0.337***	***0.014***	***0.025***
**Gender**					
Male	229	38.10 (15.85)	67.04 (10.58)	74.83 (27.72)	57.51 (19.59)
Female	70	37.71 (15.66)	70.54 (11.39)	86.44 (25.55)	63.86 (24.57)
***p-value***		***0.858***	***0.024***	***0.002***	***0.027***
**Education**					
Primary	44	29.09 (15.45)	66.76 (10.48)	83.39 (25.22)	60.68 (21.71)
Secondary	82	36.71 (15.56)	66.25 (11.21)	70.96 (30.68)	54.02 (19.62)
Higher secondary	83	36.81 (14.60)	67.32 (9.94)	78.99 (26.06)	58.8 (21.21)
Graduate & above	90	44.67 (14.66)	70.36 (11.26)	79.38 (26.55)	62.89 (21.32)
***p-value***		***0.000***	***0.064***	***0.064***	***0.045***
**Nationality**					
Asian	178	37.25 (17.27)	67.39 (11.18)	78.75 (28.43)	59.94 (21.64)
Middle east	90	38.72 (11.67)	68.36 (10.34)	73.19 (25.30)	55.55 (20.01)
African	21	36.43 (16.44)	66.07 (7.40)	81.00 (27.59)	66.19 (18.02)
Western	10	48.50 (17.00)	75.50 (13.73)	88.30 (31.48)	58.00 (20.98)
***p-value***		***0.156***	***0.109***	***0.220***	***0.153***
**Family history of stroke**					
No	227	36.60 (15.83)	67.40 (10.39)	74.65 (27.94)	55.64 (20.33)
yes	72	42.43 (14.89)	69.30 (12.17)	86.69 (24.64)	69.58 (19.60)
***p-value***		***0.006***	***0.195***	***0.001***	***0.000***
**Past medical history**					
No	26	36.15 (15.31)	64.03 (11.38)	82.46 (27.44)	60.00 (22.27)
Yes	273	38.18 (15.84)	38.22 (10.75)	77.08 (27.65)	58.90 (20.91)
***p-value***		***0.531***	***0.060***	***0.344***	***0.7992***
**Smoking history**					
Never	143	37.69 (15.80)	68.55 (10.87)	81.27 (28.75)	63.50 (21.79)
Occasionally	42	33.33 (17.34)	67.92 (11.71)	79.48 (27.48)	55.00 (18.64)
Daily	77	41.10 (14.54)	67.44 (10.86)	69.86 (24.18)	54.03 (18.16)
Stopped	37	38.11 (15.56)	66.01 (9.96)	77.00 (27.89)	56.49 (22.88)
***p-value***		***0.081***	***0.625***	***0.032***	***0.004***
**BMI**					
<20	11	27.73 (13.48)	66.82 (12.90)	85.64 (24.23)	45.45 (19.68)
20–25	71	35.07 (16.70)	67.89 (10.73)	82.18 (28.67)	61.69 (26.08)
25.1–30	93	35.97 (16.07)	66.88 (11.34)	78.80 (31.20)	62.80 (20.45)
>30	124	42.14 (14.22)	68.67 (10.44)	73.25 (23.83	55.81 (17.61)
***p-value***		***0.001***	***0.674***	***0.105***	***0.008***
**Medical insurance**					
No	181	35.88 (16.48)	67.98 (10.96)	79.58 (27.96)	59.39 (21.76
Yes	118	41.27 (14.11)	68.33 (10.71)	74.44 (26.93)	58.39 (19.83)
***p-value***		***0.004***	***0.764***	***0.116***	***0.687***

**Table 4.** Stroke knowledge, recognition, and prevention score among medication adherence and nonadherence group of participants.

**Variable**	**Medication adherence group (n = 77)**	**Medication nonadherence group (n = 222)**	***p-value***
	**Mean score (SD)**	**Mean score (SD)**	
	**(%)**	**(%)**	
Stroke knowledge	39.74 (17.05)	37.41 (15.31)	0.265
Stroke recognition	72.14 (12.34)	66.72 (9.93)	0.000
Stroke prevention	88.87 (24.75)	73.62 (27.54)	0.000

**Table 5.** Correlation between stroke awareness and medication adherence of the participants.

		**Medication adherence**	**Stroke awareness**
Medication adherence	Pearson correlation	1	0.224**
	Sig. (2-tailed)		0.000
Stroke awareness	Pearson correlation	0.224**	1
	Sig. (2-tailed)	0.000	

**Correlation is significant at the 0.01 level.
